# A comparison of performance between a deep learning model with residents for localization and classification of intracranial hemorrhage

**DOI:** 10.1038/s41598-023-37114-z

**Published:** 2023-06-20

**Authors:** Salita Angkurawaranon, Nonn Sanorsieng, Kittisak Unsrisong, Papangkorn Inkeaw, Patumrat Sripan, Piyapong Khumrin, Chaisiri Angkurawaranon, Tanat Vaniyapong, Imjai Chitapanarux

**Affiliations:** 1 Department of Radiology, Maharaj Nakorn Chiang Mai Hospital, Faculty of Medicine, Chiang Mai University, Chiang Mai, 50200 Thailand; 2 Global Health and Chronic Conditions Research Group, Chiang Mai, 50200 Thailand; 3 Department of Computer Science, Faculty of Science, Chiang Mai University, Chiang Mai, 50200 Thailand; 4 Research Institute for Health Sciences, Chiang Mai University, Chiang Mai, 50200 Thailand; 5 Department of Family Medicine, Faculty of Medicine, Chiang Mai University, Chiang Mai, 50200 Thailand; 6 Neurosurgery Division, Department of Surgery, Faculty of Medicine, Chiang Mai University, Chiang Mai, 50200 Thailand

**Keywords:** Medical research, Neurology

## Abstract

Intracranial hemorrhage (ICH) from traumatic brain injury (TBI) requires prompt radiological investigation and recognition by physicians. Computed tomography (CT) scanning is the investigation of choice for TBI and has become increasingly utilized under the shortage of trained radiology personnel. It is anticipated that deep learning models will be a promising solution for the generation of timely and accurate radiology reports. Our study examines the diagnostic performance of a deep learning model and compares the performance of that with detection, localization and classification of traumatic ICHs involving radiology, emergency medicine, and neurosurgery residents. Our results demonstrate that the high level of accuracy achieved by the deep learning model, (0.89), outperforms the residents with regard to sensitivity (0.82) but still lacks behind in specificity (0.90). Overall, our study suggests that the deep learning model may serve as a potential screening tool aiding the interpretation of head CT scans among traumatic brain injury patients.

## Introduction

Among common neurological problems, traumatic brain injury (TBI) is one of the most prevalent and poses one of the most important burdens on public health^1^. A head computed tomography (CT) scan, an effective non-invasive modality, is almost always the first-line investigation of acute TBI, owing to the widespread availability for the procedure and also the short acquisition time. CT scans have the ability to detect intracranial hemorrhage (ICH), mass effect, and associated complications. As a result, patients requiring emergency neurosurgical intervention can be identified rapidly^2^.

Due to the emergency nature of trauma, doctors need to obtain and interpret CT scans as quickly as possible. This is especially important in the case of head injuries, where timely treatment can avoid cognitive and physical disability. Emergency physicians and neurosurgeons must decide whether to plan operative or conservative treatment for the patient. The potential subtypes of ICH that may necessitate surgical intervention include intraparenchymal hemorrhage (IPH), subdural hemorrhage (SDH), and epidural hemorrhage (EDH)^3^. Rapid trauma response systems, including the availability of CT scans and adequate personnel, are required to prevent the possibly long-lasting effects of secondary brain injury and enhance patient outcomes^4^.

However, the number of trained radiologists or even radiology trainees available to interpret the CT scans has often been limited, resulting in significant delays in analyzing and reporting results^5,6^. Thus, in resource-limited settings, treatment planning before the formal radiology report may result in misinterpretation and inappropriate clinical management.^7,8^.

A promising solution to tackle this problem is the usage of Artificial Intelligence (AI). Many studies have used deep learning methods to assist in the diagnosis of diseased, oncologic, and traumatic patients^9–12^. An automated ICH detection and classification tool may assist residents or clinicians when medical radiology experts are not immediately available^13,14^. Deep learning models have also been implemented to detect ICH and even assess mass effects from ICH in both retrospective and prospective studies^15–17^. The majority of the studies^17–21^ focused on the evaluation of diagnostic accuracy for the identification of ICH and classification into each ICH subtype using algorithms, but, to date, the accuracy of the detection of ICH into specific intracranial locations has not been well evaluated.

Recently, the authors of this study developed a deep learning model for segmenting SDH, EDH, and IPH^22^. The model proposed outperformed segmentation performance with a higher dice score when compared to reports in previously published literature^22^. In this study, we aim to compare the performance of the proposed deep learning segmentation model^22^ with that of radiology, emergency, and neurosurgery residents. We primarily focus on the detection of IPH, SDH, and EDH, as these subtypes are usually the ones being evaluated in identifying and selecting TBI patients for neurosurgical intervention^3^.

## Material and methods

### Development of the deep learning model

The deep learning model we investigated in this study was proposed in our prior published study^22^. The model is a variation of the DeepMedic^23^ model that has the ability to segment SDH, EDH, and IPH on a CT scan. Its architecture consists of four parallel pathways that process the input at different resolutions and two fully connected layers. It obtains a 2-channel voxel extracted from the subdural and bone windows of a brain CT scan as the input. All voxels in a CT scan were processed by the model to generate segmentation results with the value of each pixel as the class label of the hemorrhage type where the pixel was located. After the segmentation results were produced by the deep learning model, the regions of SDH and EDH with their major axis of less than 5 mm were considered as noise and removed from the segmentation result. The final results were drawn on the input CT scan by assigning different colors to hemorrhage types. The model is shared via https://github.com/RadiologyCMU/Hemorrhage-DeepMedic. In this study, the deep learning model was performed without any fine-tuning. We also adopted the same data pre-processing process used in our previous work. In addition, a new dataset was used as the test dataset, which differed from the dataset used to train the model in^22^. The samples in the test dataset were randomly selected from patients who were not in the training dataset. Patients who received multiple scans, we have chosen only the first scan.

### Study cohort

Non-contrast head CT scans of adult patients aged 15 years or older suspected of head injury/trauma as the initial presentation during emergency department (ED) visits at Maharaj Nakorn Chiang Mai Hospital from January 1, 2014, to December 31, 2014, were included. Exclusion criteria included: (1) Follow-up CT studies of patients with known recent TBI; (2) Studies of patients with recent neurosurgical intervention; (3) Severe artifacts degrading study quality such as motion artifacts and metallic artifacts.

Brain CT scans were acquired with CT equipment from either of two manufacturers (Toshiba Aquilion 16 or Siemens SOMATOM Definition). Each slide was stored as a 512 × 512 pixels DICOM image. The typical image resolution of x and y is 0.4473 mm per pixel.

The number of image slices per patient may vary between 80 and 115, depending on factors such as the size of the patient's head, with a fixed separation distance between slices of 1.5 mm. With these criteria, a total of 300 head CT studies from different subjects were finally included, 166 studies were categorized under the intracranial hemorrhage (ICH) group, while 134 studies were classified under the non-ICH group. After thorough review and annotation from consensus of two experienced neuroradiologists out of the 300 head CT studies, 171 were identified as lesions of IPH (27.01%), 356 lesions of SDH (56.24%), and 106 lesions of EDH (16.75%). Detailed data describing locations of lesions are shown in Table 1.

**Table 1:** Type and distribution of ICHs. Abbreviations: ICH, intracranial hemorrhage; IPH, intraparenchymal hemorrhage; EDH, epidural hemorrhage; SDH, subdural hemorrhage.

Type of ICH	Number of lesions
**IPH (lesions)**	171
Locations	
Right hemisphere	84
Left hemisphere	67
Right cerebellar convexity	2
Left cerebellar convexity	1
Right Deep Nuclei	8
Left Deep Nuclei	3
Midbrain	5
Pons	1
**SDH (lesions)**	356
Locations	
Right cerebral convexity	74
Left cerebral convexity	75
Falx cerebri	100
Tentorium cerebelli	99
Right cerebellar convexity	5
Left cerebellar convexity	3
**EDH (lesions)**	106
Locations	
Right cerebral convexity	48
Left cerebral convexity	49
Right cerebellar convexity	3
Left cerebellar convexity	5
Vertex	1

### Classification of ICH by deep learning model

**Figure 1:**
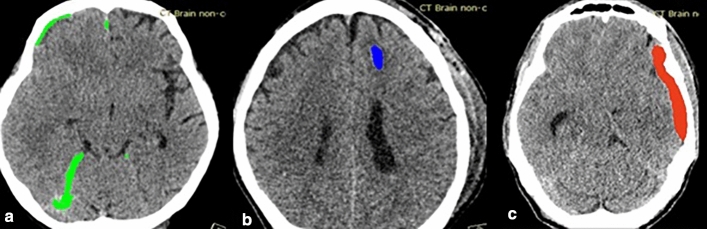
ICH Segmentation with Different Colors Referring to Different Types of ICH. (**a**) SDHs are colored in green along the right cerebral convexity, falx cerebri, and tentorium cerebelli, equivalent to 3 locations of SDHs; (**b**) IPH is colored in blue at the left frontal lobe, equivalent to the left cerebral hemisphere, and is counted as 1 location; (**c**) EDH at the left cerebral convexity is colored in red.

The deep learning model was then used to identify negative and positive studies. In the positive studies, the subtypes of ICH of concern were classified and segmented. The deep learning model segmented the area of ICH with different color maps at specific locations indicating different subtypes and locations of ICH. Correct segmentation means coloring true ICH in the expected locations, as in Fig. 1. The true location of ICH but incorrect color regarding ICH subtypes were considered false interpretations.

### Classification of ICH by residents

Four radiology residents** (**three junior radiology residents and one senior radiology resident) and four non-radiology residents (two senior emergency medicine residents and two senior neurosurgery residents) were recruited for the study. These three areas of specialist were chosen as the residents attached to these areas of expertise were most likely to be those making the initial CT interpretation at the emergency department. Blinded to the original CT results, all eight residents were independently required to interpret all head CT scans solely from the CT series consisting of a 1.5-mm thick slice in the axial plane. The residents could manually adjust the width and level of the window for each scan during interpretation.

**Figure 2:**
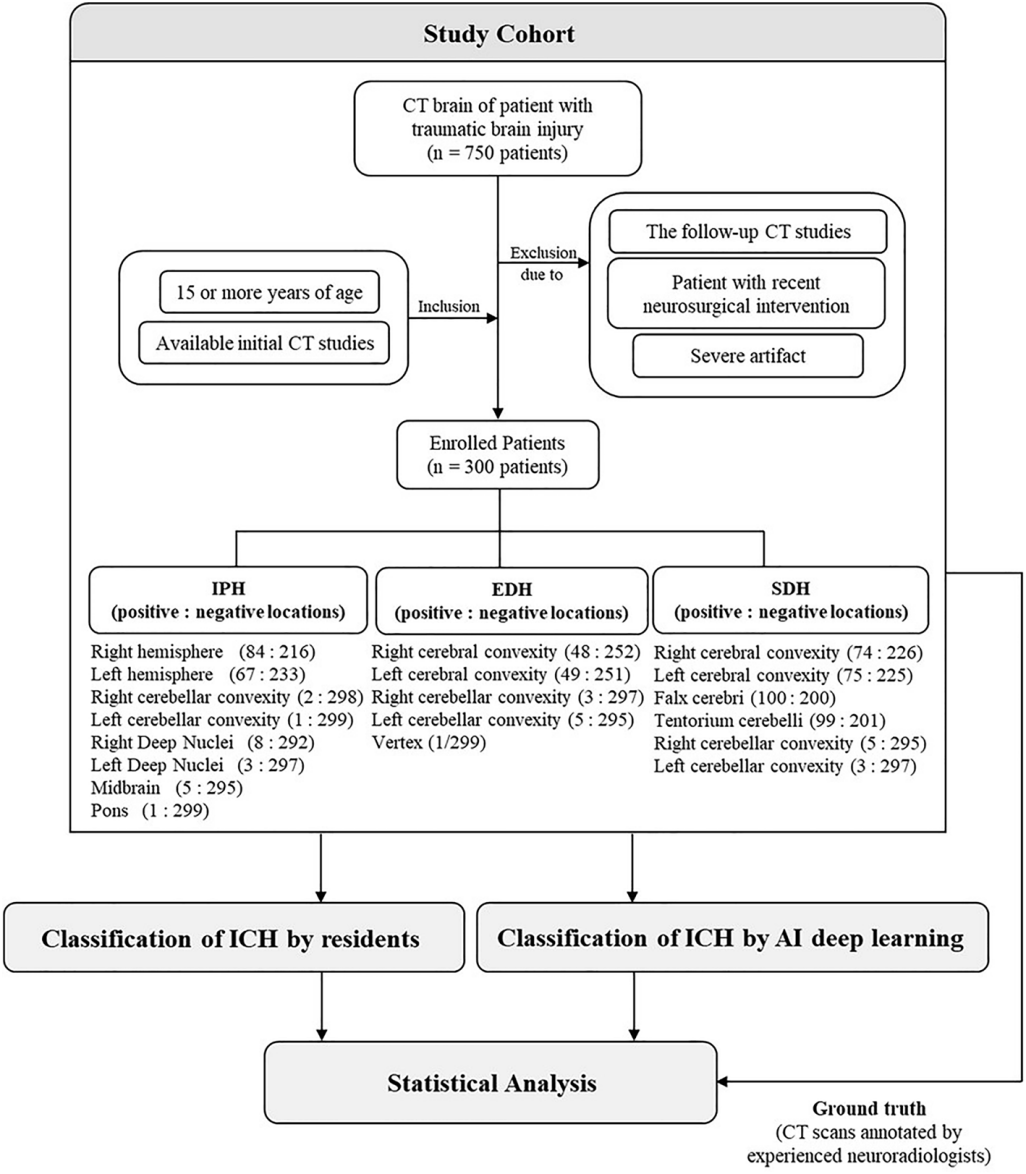
Flowchart of this study.

A record form was created consisting of multiple-choice checkboxes regarding the location of each ICH subtype. The locations for IPH were: (1) Right cerebral hemisphere; (2) Left cerebral hemisphere; (3) Right cerebellar hemisphere; (4) Left cerebellar hemisphere; (5) Right deep nuclei; (6) Left deep nuclei; (7) Midbrain; (8) Pons. Deep nuclei encompassed either the caudate nucleus, lentiform nucleus, or thalamus. In the case of SDH, the locations could be: (1) Right cerebral convexity; (2) Left cerebral convexity; (3) Falx cerebri; (4) Tentorium cerebelli; (5) Right cerebellar convexity; (6) Left cerebellar convexity. For EDH, the locations included: (1) Right cerebral convexity; (2) Left cerebral convexity; (3) Right cerebellar convexity; (4) Left cerebellar convexity; (5) Vertex. If no ICH locations were identified in IPH, SDH, nor EDH these were classed as negative-ICH. A flowchart methodology of this study is available in the Fig. 2.

### Statistical analysis

**Figure 3:**
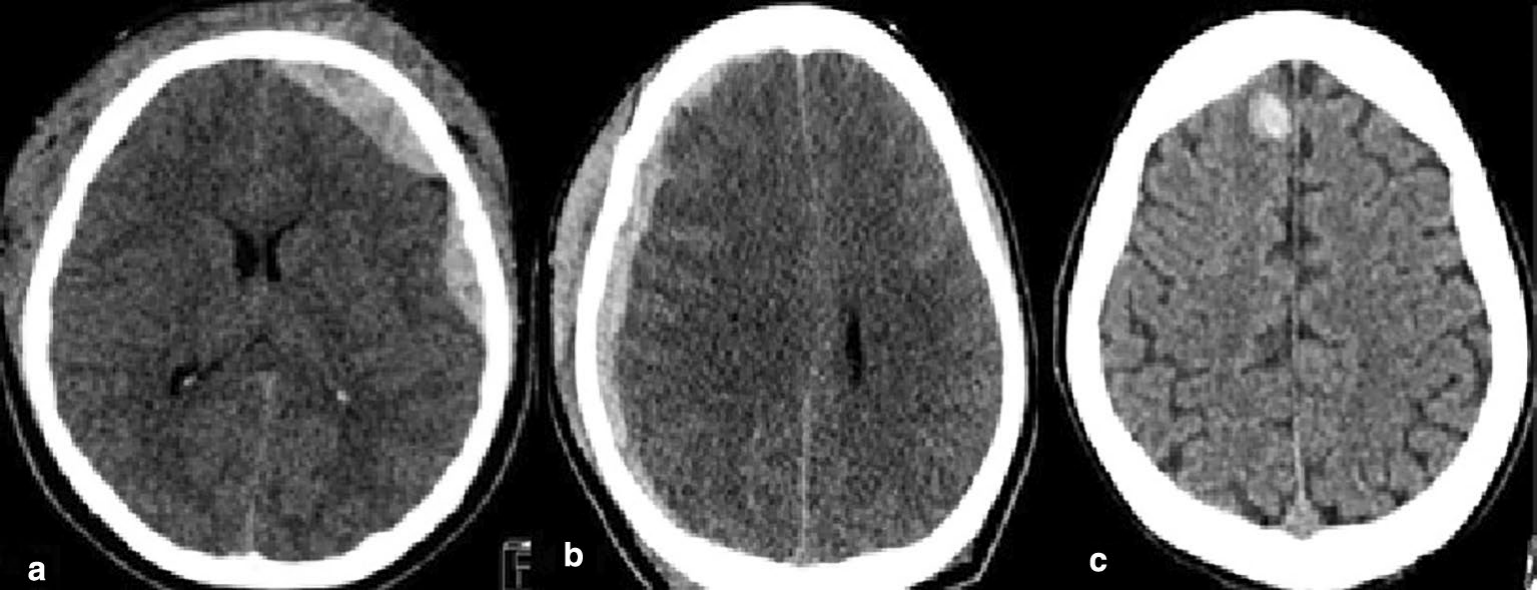
Identification and Localization of ICH: (**a**) EDH at left cerebral convexity, (**b**) SDH at right cerebral convexity, (**c**) IPH at right cerebral hemisphere.

In most cases, there were more than one ICH subtype and/or multiple lesions of the same subtype. The algorithm or trainee residents would have to correctly identify all ICH subtypes and a correct location was considered as “detected”. If any ICH remained undetected or was mis-identified either by subtype or location, it was counted as “missed”. The imaging of ICH subtypes in certain locations is presented in Fig. 3. We evaluated the performance of the algorithm and trainees using statistical metrics, including accuracy, sensitivity, and specificity, using the Python package scikit-learn. A significant difference was considered when *p* < 0.05.

### Informed consent and ethical approval

This study was approved by the Research Ethics Committee of the Faculty of Medicine, Chiang Mai University (No.423/2021). Informed consent was obtained from all participants. All methods were performed in accordance with the relevant guidelines and regulations.

## Results

We evaluated the performance of the deep learning model and compared it to that of the eight training residents in the classification and localization of ICHs based on the individual locations occupied by specific types of ICH. The accuracy, sensitivity, and specificity of the algorithm and residents are displayed in Table 2. In terms of ICH detection and localization, the model achieved an accuracy of 0.89 with a sensitivity, and specificity of 0.82 and 0.90, respectively. Overall, four radiology residents achieved accuracy, sensitivity, and specificity of 0.96 ± 0.00, 0.74 ± 0.04, and 0.99 ± 0.01, whereas four non-radiology residents scored values of 0.94 ± 0.01, 0.61 ± 0.08, and 0.99 ± 0.00, respectively.

**Table 2:** Location-level performance of the algorithm, three junior radiology residents, a senior radiologist, two emergency residents, and two neurosurgery residents presented as accuracy, sensitivity, and specificity. Abbreviations: ICH, intracranial hemorrhage; IPH, intraparenchymal hemorrhage; EDH, epidural hemorrhage; SDH, subdural hemorrhage.

Hemorrhage	Interpreters	Accuracy	Sensitivity	Specificity
ICH	Model	0.89	0.82	0.90
	Junior radiology resident 1	0.96	0.78	0.98
	Junior radiology resident 2	0.96	0.70	0.99
	Junior radiology resident 3	0.96	0.71	0.99
	Senior radiology resident	0.96	0.78	0.98
	Radiology residents	0.96 ± < 0.01	0.74 ± 0.04	0.99 ± 0.01
	Senior EM resident 1	0.95	0.70	0.98
	Senior EM resident 2	0.94	0.56	0.99
	Senior NS resident 1	0.94	0.52	0.99
	Senior NS resident 2	0.95	0.64	0.99
	Non-radiology residents	0.94 ± 0.01	0.61 ± 0.08	0.99 ± < 0.01
IPH	Model	0.93	0.83	0.94
	Junior radiology resident 1	0.97	0.78	0.99
	Junior radiology resident 2	0.98	0.79	0.99
	Junior radiology resident 3	0.98	0.77	0.99
	Senior radiology resident	0.98	0.85	0.99
	Radiology residents	0.98 ± < 0.01	0.80 ± 0.04	0.99 ± < 0.01
	Senior EM resident 1	0.96	0.79	0.97
	Senior EM resident 2	0.97	0.65	0.99
	Senior NS resident 1	0.97	0.73	0.98
	Senior NS resident 2	0.97	0.68	0.99
	Non-Radiology residents	0.96 ± < 0.01	0.71 ± 0.06	0.98 ± 0.01
EDH	Model	0.91	0.72	0.93
	Junior radiology resident 1	0.97	0.74	0.99
	Junior radiology resident 2	0.97	0.61	1.00
	Junior radiology resident 3	0.98	0.76	0.99
	Senior radiology resident	0.97	0.71	0.99
	Radiology residents	0.97 ± < 0.01	0.71 ± 0.07	0.99 ± < 0.01
	Senior EM resident 1	0.97	0.71	0.99
	Senior EM resident 2	0.96	0.55	0.99
	Senior NS resident 1	0.96	0.52	0.99
	Senior NS resident 2	0.97	0.74	0.99
	Non-Radiology residents	0.97 ± 0.01	0.63 ± 0.11	0.99 ± < 0.01
SDH	Model	0.82	0.85	0.82
	Junior radiology resident 1	0.93	0.79	0.96
	Junior radiology resident 2	0.93	0.69	0.99
	Junior radiology resident 3	0.92	0.67	0.99
	Senior radiology resident	0.92	0.76	0.96
	Radiology residents	0.93 ± < 0.01	0.73 ± 0.06	0.97 ± 0.01
	Senior EM resident 1	0.91	0.66	0.98
	Senior EM resident 2	0.89	0.51	0.98
	Senior NS resident 1	0.88	0.42	0.99
	Senior NS resident 2	0.91	0.59	0.99
	Non-Radiology residents	0.90 ± 0.02	0.54 ± 0.11	0.98 ± 0.01

Regarding the detection and localization of each ICH subtype, the deep learning model was the most sensitive in detecting SDH (sensitivity = 0.85). The sensitivities for detecting IPH and EDH were 0.83 and 0.72, respectively. The radiology residents performed similarly well to the model in the sensitivity of each ICH subtype. The sensitivity for IPH was 0.80, 0.71 for EDH, and 0.73 for SDH. The neurosurgery and emergency medicine residents had lower performance scores in detecting each ICH subtype, with a sensitivity of 0.71 for IPH detection, 0.63 for EDH, and 0.54 for SDH. The model had an overall higher sensitivity for ICH detection than the average performance of training residents across all ICH subtypes (*p* < 0.05).

**Figure 4:**
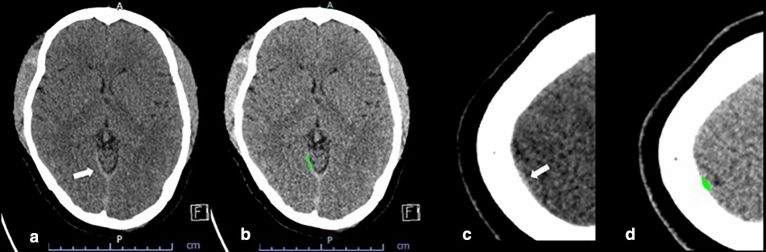
SDH Along Right Tentorium Cerebelli (**a**) are correctly colored green by the algorithm (**b**). Another case with subtle right cerebral convexity SDH (**c**) was also detected by the algorithm and colored in green (**d**).

**Figure 5:**
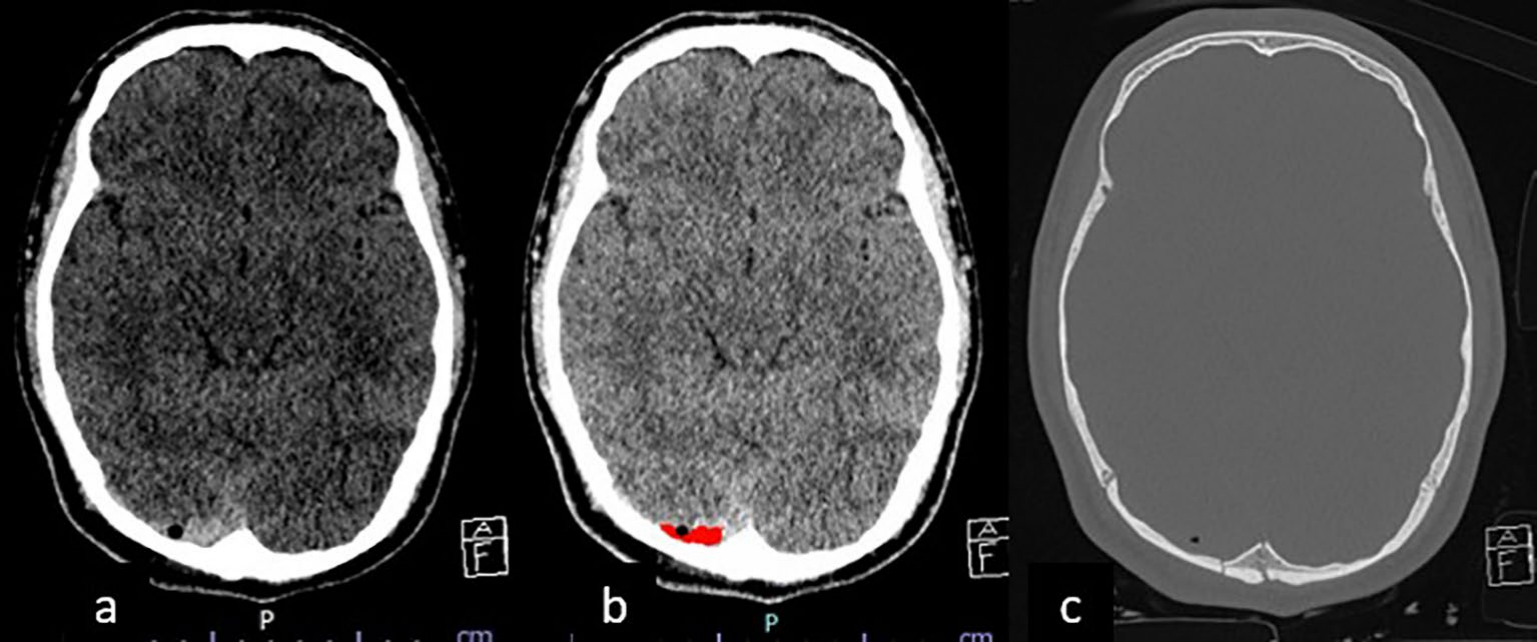
Missed EDH at Right Cerebral Convexity (**a**) is correctly colored by the algorithm in red (**b**) with associated skull fracture demonstrated on bone window image (**c**).

**Figure 6:**
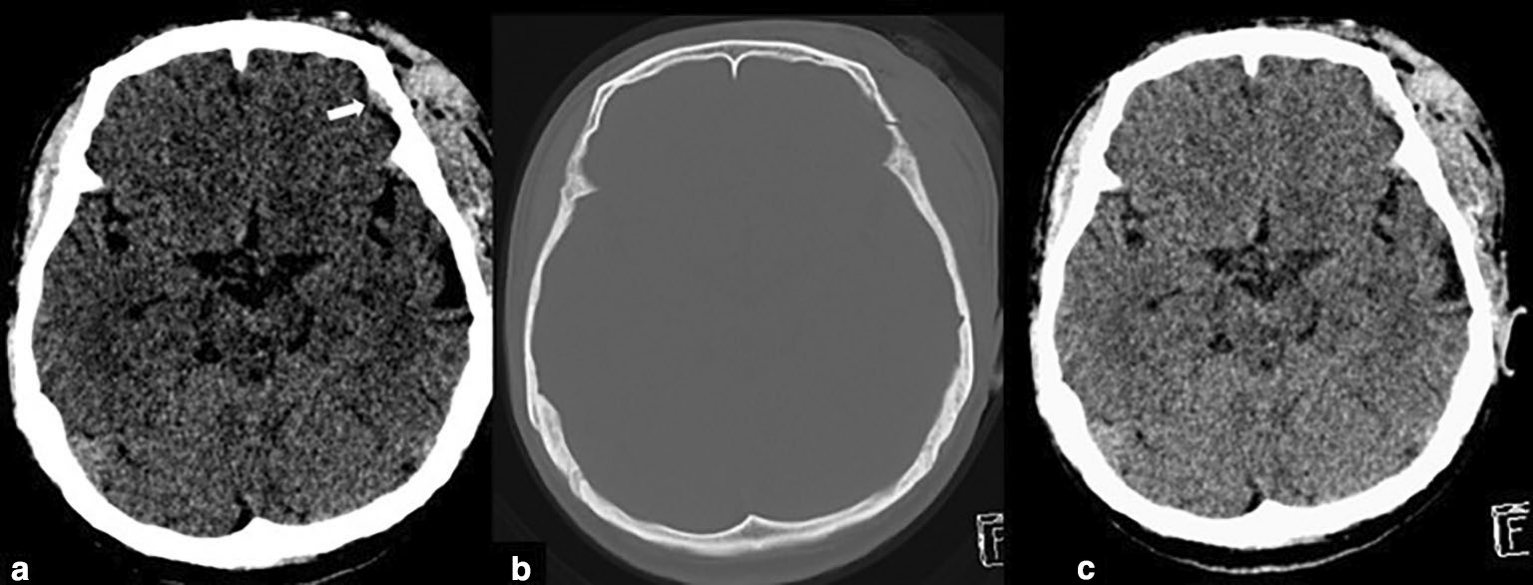
The CT scan of the brain shows a small left frontal convexity EDH (white arrow) in narrowed window setting (**a**) and associated skull fracture in the bone window setting (**b**). The post-processing image reveals that the EDH has not been annotated by the algorithm **c**).

In several cases, the deep learning model detected subtle SDH when this could not be detected by any of the residents. Most of these cases were studies containing thin SDHs either along the tentorium cerebelli or the cerebral convexities. Some of these cases had various hemorrhagic subtypes resulting in small hemorrhages being overlooked. Three subtle EDHs were missed by radiology residents, and five subtle EDHs were missed by non-radiology residents. However, all of these EDHs were picked up by the algorithm. These cases are illustrated in Figs. 4 and 5. Only two cases of small EDHs were missed by the deep learning but were able to be detected by all residents (Fig. 6).

**Figure 7:**
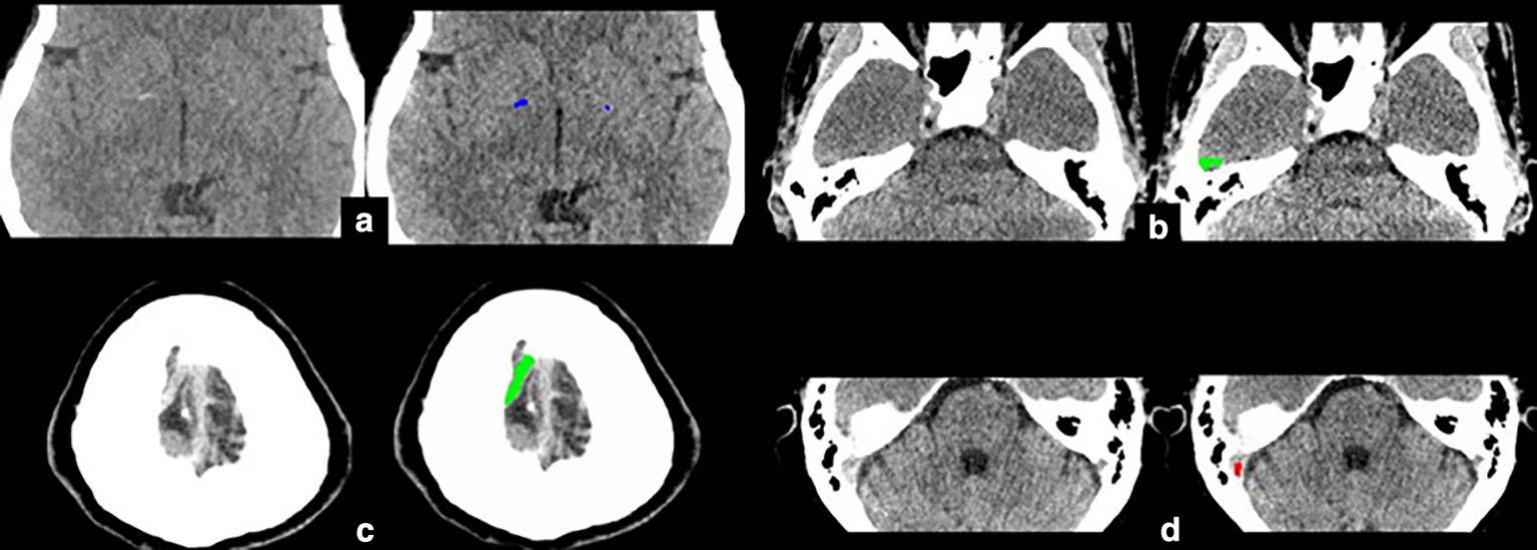
Demonstrating the misinterpretation of basal ganglia calcification by the algorithm (**a**), beam hardening artifact (**b**), cortical vein (**c**), and sigmoid sinus (**d**) as ICHs.

The specificities for ICH detection and subtypes of ICH by the deep learning and residents were relatively high. The overall specificity for ICH detection by the algorithm was 0.90, while the specificities for ICH detection by radiology and non-radiology residents were both 0.99. Specificity values for each subtype of ICH varied between 0.82 and 0.90 in the case of the deep learning, while the specificity values varied between 0.97 and 0.99 for the residents for each ICH subtype (Table 2). There were many cases in which the deep learning model overdiagnosed, usually involving basal ganglia calcification, beam hardening artifacts, dense cortical veins, and dural venous sinuses being interpreted as hemorrhage. Some of these studies are presented in Fig. 7.

## Discussion

Our results demonstrate non-inferior diagnostic accuracies in ICH detection of the deep learning model compared to residents (*p* > 0.05). In terms of sensitivity, the model yielded noticeably higher overall sensitivity values for ICH detection across nearly all subtypes compared to the residents (*p* < 0.05). Nonetheless, specificity of the model still falls behind that of the residents.

In our study, we determined the accuracy of the algorithm by evaluating the frequency of correct and incorrect ICH detection from each location. The deep learning model achieved high accuracy of overall ICH detection, and IPH, EDH, and SDH detection (0.89, 0.93, 0.91, and 0.82). The reported diagnostic performance of deep learning models in prior literature were variable, with accuracy values ranging from 0.70 to 0.94^13^. Similar studies based on convolutional neural networks yielded approximate accuracy values from 0.81 to 0.90^20,24^. The variations in accuracy are likely due to the classification methods among different models and ways of measurement for ICH segmentation which might not allow direct comparison between studies.

Few studies have compared the diagnostic performance between algorithms and trainee residents. Ye et al. demonstrated superior performance of the deep learning neural network. They concluded that their algorithm was fast and accurate, indicating its potential role in assisting junior radiology residents in reducing misinterpretation of head CT scans^15^. However, they primarily focused on ICH detection and classification, while our study also stresses the importance of identifying both the subtype and correct location.

Although our deep learning model demonstrated high specificity, it still lags behind that of the residents. From a review of the lesion segmentation by the algorithm, many non-ICH findings had been misinterpreted as ICH. This was possibly because identification of ICH required the region be of higher attenuation or higher Hounsfield unit (HU) than surrounding normal brain parenchyma. Other findings with high HU, such as basal ganglia calcification, beam hardening artifacts, dense cortical veins, and dural venous sinuses, were misclassified as hemorrhage. This is concordant with prior results in that common AI overcalls are calcification and beam-hardening artifacts^25,26^. While radiologists and clinicians gain experience in identifying actual ICHs, AI analysis software must also be trained to recognize these ICH mimickers.

In terms of sensitivity, the deep learning model yielded noticeably higher overall sensitivity values for ICH detection across nearly all subtypes compared to the residents (*p* < 0.05). Moreover, the deep learning model could detect subtle hemorrhages missed by residents, such as thin SDH and small EDH. Waite et al. suggest that in their study of interpretative errors in radiology perceptual errors account for 60%-80% of radiological errors^27^. Since perception and detection are the initial phases in image interpretation, an error in this phase can abruptly terminate the diagnostic process and result in a mistaken (false-negative) diagnosis. Perceptual errors in radiology have been linked to a variety of causes, including fatigue of the interpreter and the increased pace of interpretations^28^. The error rate could also change depending on the time of day the interpretation was given, and long and overnight shifts are associated with increased rates of inaccuracy, according to previous studies^29,30^. More crucially, in approximately 1% of cases, the diagnostic error results in incorrect or inadequate patient management^31,32^. This reinforces the vital role of a deep learning model as a potential screening tool in emergency cases requiring rapid ICH detection or as an assistant for trainee residents in generating emergency CT reports. Similar work has been done using an active automated tool to detect acute intracranial conditions, including hemorrhage, reprioritizing study, and notifying radiologists if ICH was identified as present. Thus, these resulted in significant reductions in diagnostic waiting time with high sensitivity with regard to detection^33,34^.

Different sensitive and specificity tradeoff is likely to be different depending on their role in the care pathway. If primary role of the operator, whether it be the radiologist, emergency physician or AI, is to screen/inform or refer for appropriate interventions, due to the serious and urgent nature of TBI and importance of timely surgical intervention for some subtypes of TBI, it may be better to set a high sensitivity and allow more false positives (lower positive predictive value). As a reference, the American College of Surgeons Committee on Trauma has set the national benchmark for field triage at ≥ 95% for sensitivity and ≥ 50% specificity^35^. However, if the primary role of the operator, whether it be the radiologist, neurosurgeon or AI, is to decide the definitive treatment on whether or not to perform neurosurgery, it would be better to set a high specificity.

Following this argument, outcomes of this study suggest that the deep learning model may be a useful screening tool for the detection and localization of ICH from CT scans in the cases of traumatic brain injury in the majority of emergency departments (ED), in particular where there may not be 24-hour coverage of trained radiologists. However, confirmation by trained personnel is required before definitive surgical treatment plans can be made. This is particularly the case in low- and middle-income settings when emergency physicians and neurosurgeons frequently evaluate emergency computed tomography (CT) scans without support from trained radiologists.

There are three main limitations to our study. First, the performance evaluation is done by scoring the detected ICH according to crude locations, which sometimes represent a wide and less specific area within the cranium. For example, hematoma in the cerebral hemisphere could be in either the frontal, parietal, temporal, or occipital lobes. Multiple separated hematomas may be present in different locations within the same hemisphere. If only one hematoma is segmented or identified in the setting of multiple discrete hematomas confined within the recording area (e.g., a hemisphere), this will be erroneously considered as all hematomas being detected when the remaining hematomas have been missed. However, unlike a previous study^15^, ours is one of the few that has attempted to match the type of ICH and their precise locations with performance comparisons to training residents rather than simply detecting ICH subtype alone. The second limitation is the sample size; with only 300 cases of head CT studies, the study may not have the same level of statistical power in defining the exact diagnostic performance of the deep learning model in comparison to some other studies in the literature^16,17,36^. In addition, all the subjects were limited to 15 or more years of age. In future studies, it would be useful if the data set were expanded to include all age groups. Lastly, we did not validate the deep learning model with the external dataset (e.g. from another hospital or geographical area). Samples in the test dataset were collected from the same hospital and CT machines used for the training dataset. This limitation needs to be addressed in future validation.

## Conclusion

In conclusion, our study is one of the first to validate the efficacy of the role of the deep learning model for ICH detection and localization by comparing the level of diagnostic accuracy with radiology, emergency medicine, and neurosurgery residents. Based on the results, our study highlighted the potential use of AI as a useful intracranial hemorrhage screening tool in traumatic brain injury patients. However, its slightly lower specificity and tendency to misinterpret some benign lesions with high attenuation into hemorrhage remain an issue to be addressed. Further model training with a larger data set and a larger sample size is expected to improve the overall capability of our deep learning model in a real clinical setting.

## Data Availability

The datasets generated during and/or analysed during the current study are available from the corresponding author on reasonable request.
